# Exposure to Heated Tobacco Products Aerosol Causes Acute Stress Responses in the Lung of Mouse

**DOI:** 10.3390/antiox11122329

**Published:** 2022-11-25

**Authors:** Shin Koike, Kohei Sato, Marie Sawa, Yohei Inaba, Kenji Hattori, Kazuhiko Nakadate, Akira Ushiyama, Yuki Ogasawara

**Affiliations:** 1 Department of Analytical Biochemistry, Meiji Pharmaceutical University, 2-522-1 Noshio, Tokyo 204-8588, Japan; 2 Department of Environmental Science, Meiji Pharmaceutical University, 2-522-1 Noshio, Tokyo 204-8588, Japan; 3 Department of Environmental Health, National Institute of Public Health, 2-3-6 Minami, Saitama 351-0197, Japan; 4 Department of Basic Science, Educational and Research Center for Pharmacy, Meiji Pharmaceutical University, 2-522-1 Noshio, Tokyo 204-8588, Japan

**Keywords:** heated tobacco product, oxidative stress, ER-stress, ATF4, HO-1, GSH

## Abstract

In the present study, we evaluated the acute response of mice exposed to IQOS aerosol, a brand-name heated tobacco product (HTP), in the lung tissue. First, the thiobarbituric acid-reactive substances (TBA-RS) value was measured as an index to assess oxidative stress, and a significant increase was observed after exposure, followed by a significant increase in the total lung GSH concentration. The stress responses induced by IQOS aerosols was then analyzed by focusing on the changes in Nrf2 and ATF4, which are transcription factors that induce the expression of genes involved in GSH biosynthesis or metabolism. Although Nrf2 activation was not observed, significant accumulation of ATF4 in the nuclear fraction was noted three hours after exposure to IQOS aerosols. Upon an examination of changes in factors in the GSH biosynthetic system, a significant increase in cystine concentration in the lung tissue was measured, and an increase in xCT expression level was observed in the cell membrane fraction three–six hours after IQOS exposure. Furthermore, characteristic changes in HO-1, a stress-response protein regulated by ATF4, was discovered six hours after IQOS exposure. Moreover, analysis of the upstream ATF4 regulatory system revealed that phosphorylation of eIF2α was enhanced in the lung cytoplasmic fraction three hours after exposure to IQOS aerosols. These findings suggest that ER stress might be induced as an early response to IQOS aerosol exposure, accompanied by the activation of the eIF2α-ATF4 axis. These intracellular changes have also been reported after exposure to combustible cigarette smoke. Thus, the acute response found in the lungs of mice in the present study demonstrate that the inhalation of aerosols from IQOS elicits a biological response similar to that of combustible cigarette smoke. In conclusion, our results provide evidence that the biological effects of HTPs, such as IQOS, cannot be ignored in the lungs.

## 1. Introduction

Heated tobacco products (HTPs) are an alternative to conventional combustible cigarettes, thus reducing the number of hazardous substances ingested.

Philip Morris International developed HTP, also called a “Heat-not-Burn” product, that is currently marketed under the brand name “IQOS”. It was launched in 2014 in Japan and Italy [1] and has expanded to 69 countries [2]. The tobacco industry claims that HTPs are less harmful to humans than combustible cigarettes. However, there are only a few reports from public research institutions that do not receive research funding from the tobacco industry. Therefore, the World Health Organization has declared that there is no evidence to suggest that reduced exposure to chemicals means that they are less harmful, nor does it translate into reduced risk in humans [3]. Indeed, our research and studies by third-party institutions have shown that the content of harmful substances (nicotine, tar, carbon monoxide, nitrosamines, etc.) is reduced [4]; however, certain chemicals are present at higher concentrations in the HTP aerosols than in conventional combustible cigarettes [5,6,7]. Notably, the amount of hazardous substances and their effects are not always proportional, and even infrequent smoking may increase the risk of disease [8]. Therefore, even if toxic chemicals in HTPs are reduced compared to combustible cigarettes, it remains unclear whether their health effects are also reduced.

Although the tobacco industry has produced reports on the health effects of HTPs [9,10], studies on this topic from other sources are limited in number and quality. A recently published systematic review of HTPs concluded that there were too few studies and reported cases to assess their health effects [11,12]. Accordingly, it is necessary to gather evidence from additional epidemiological, in vitro, and in vivo studies [3].

Recently, we observed significant oxidative effects after exposing lung epithelial cells to an aerosol extract of HTPs [13]. Furthermore, we developed an animal exposure apparatus customized to HTP aerosols that can be used to analyze the biological effects of IQOS exposure [14]. We immediately performed the first study to evaluate the short-term effects of IQOS inhalation. When mice were exposed to IQOS aerosols for one or two days using the developed animal exposure apparatus, we observed a decrease in the concentration of GSH in alveolar macrophages and an increase in the percentage of oxidized GSH (GSSG) in lung tissues. In addition, the concentration of the inflammatory cytokines IL-6 and GM-CSF significantly increased in the plasma after IQOS exposure [15]. These results indicate that transient exposure to IQOS aerosols may cause oxidative stress and inflammation in lung tissues.

In this study, we exposed mice to IQOS aerosols using an animal exposure apparatus and evaluated the acute biological effects of IQOS aerosol inhalation in detail, focusing on the stress responses in a broader sense in the mouse lung. This study clearly indicates that even transient exposure to HTP aerosols has similar biological effects to those seen when smoking combustible cigarettes. Thus, the long-term inhalation of IQOS may have adverse health effects.

## 2. Materials and Methods

### 2.1. Animals

Seven-to nine-week-old male C57BL/6N mice (Japan SLC, Inc., Shizuoka, Japan) were housed in individually ventilated cage systems (Super Mouse 1400^TM^; Lab Products Inc., Seaford, DE, USA) under a 12 h light/dark cycle with free access to water and standard chow, controlled temperature (23 ± 1 °C), and 50 ± 15% humidity. All experimental protocols were approved by the Committee for Animal Experiments at the National Institute of Public Health (protocol number 31-003) and complied with all the guidelines and laws for animal experiments in Japan.

### 2.2. IQOS Aerosol Exposure Apparatus and Protocol

The mice were immobilized in tube-shaped retainers and placed at the exhaust port of the aerosol exposure apparatus for HTPs [15]. The mice were then exposed to IQOS aerosols from five Marlboro Heatsticks (REGULAR) per day, and lung tissues were prepared after perfusion with PBS to remove blood at 1, 3, 6, and 24 h after exposure. To obtain lung tissues from mice for comparison (sham control), similar operations were performed without aerosol inhalation from heatsticks. The puff profile, established by the Health Canada Intense smoking regimen, was defined as a puff volume of 55 mL, puff duration of 2 s, and puff interval of 30 s until the next puff, and 100% blockage of the ventilation holes in a filter with Mylar adhesive tape. The total retention time was kept as short as possible to minimize restraint stress.

### 2.3. Preparation of Tissue Extracts

Whole-lung extracts were prepared from male mice lungs with nine volumes of ice-cold buffer (pH 7.4:5 mM phosphate buffer containing a complete protease inhibitor cocktail (Nacalai Tesque, Kyoto, Japan)) using Biomasher SP (Nippi Inc., Tokyo, Japan) for the thiobarbituric acid-reactive substances (TBA-RS) assay. Cytoplasmic and nuclear extracts were prepared using NE-PER™ nuclear and cytoplasmic extraction reagents (the protease and phosphatase inhibitors were added to CER I and NER) (Thermo Scientific, Rockford, IL, USA) using Biomasher II, according to the manufacturer’s instructions. Membrane extracts were prepared using the Mem-PER™ Plus Membrane Protein Extraction Kit (Thermo Scientific, Rockford, IL, USA) and Biomasher II according to the manufacturer’s instructions.

### 2.4. Determination of TBA-RS Concentration in Whole Lung Tissue

The concentration of TBA-RS in whole lung tissues was determined using the Cayman TBA-RS assay kit (#700870). TBA-RS was measured colorimetrically at 535 nm using a microplate reader (Perkin Elmer, EnSpire, Waltham, MA, USA). The TBA-RS concentration was expressed as μmol/g wet weight.

### 2.5. Determination of GSH and Cystine Concentration in Whole Lung Tissue

GSH and cystine concentrations in mouse whole-lung extracts were measured using a previously described method with minor modifications, using HPLC with fluorescence detection (HPLC-FL) [16,17]. Four times the volume of 5% sulfosalicylic acid was added to the whole lung tissue and the tissue was subsequently homogenized using a BioMasherII (Nippi Inc., Tokyo, Japan). The homogenates were centrifuged at 12,000× *g* for 10 min at 4 °C, and the supernatant was collected. To determine reduced GSH, the supernatant was diluted with 3 times the volume of 0.2 M borate buffer (pH 10.5) and incubated with 4 mM 4-(Aminosulfonyl)-7-fluoro-2,1,3-benzoxadiazole (ABD-F) at 60 °C for 10 min, which specifically reacts with SH residues to form a fluorescent adduct. To determine the concentration of GSH and cystine, the reducing agent, tris (2-carboxyethyl) phosphine, was added to the ABD-F solution. After centrifugation at 15,000× *g* for 5 min, the supernatant was used to measure GSH levels by HPLC-FL. Ten microliters of the resulting mixture were injected into a C18 column (Cosmosil, 4.6 × 250 mm, Nacalai Tesque. Inc., Kyoto, Japan) pre-equilibrated with the mobile phase solution, which consisted of 0.1 M acetate buffer (pH 3.8): acetonitrile (92:8). A flow rate of 1.0 mL/min was used, with a running time of 20 min. The retention times and peak areas were monitored at the excitation and emission frequencies of 380 and 510 nm, respectively. The concentration of GSSG was calculated using the following equation: [GSSG] = [total GSH] − [reduced form of GSH].

### 2.6. Western Blot Analysis

Nuclear, cytoplasmic, and membrane extracts from mouse whole lungs were boiled for 5 min at 90 °C in SDS sample buffer with a reducing reagent (Nacalai Tesque, Kyoto, Japan). Proteins were separated using 5–20% SDS-polyacrylamide gel electrophoresis (ATTO, Tokyo, Japan) and then transferred onto an Immobilon-P polyvinylidene difluoride membrane (Millipore, Billerica, MA, USA). Blocking was performed using 4% (*w*/*v*) Block Ace (DS Pharma Biomedical, Osaka, Japan). Next, the membrane was incubated with primary antibodies against ATF4 (Cell Signaling Technology, Danvers, MA, USA), Eukariotic initiation factor (eIF)2α (Gene Tex, Irvine, CA, USA), phosphorylated (p)-eIF2α (Cell Signaling Technology), γ-GCLc (Santa Cruz Biotechnology, Dallas, TX, USA), γ-GCLm (Proteintech, Rosemont, IL, USA), HO-1 (Cell Signaling Technology), Nrf2 (Santa Cruz Biotechnology), LaminB1 (Cell Signaling Technology), and β-actin (Cell Signaling Technology). The membrane was then washed with PBS and incubated with secondary antibodies (HRP-conjugated goat anti-mouse IgG antibody or HRP-conjugated goat anti-rabbit or anti-mouse IgG antibody from Vector Laboratories). Protein bands were detected using Crescendo Western Reagents (Millipore, Billerica, MA, USA) or SuperSignal™ West Femto Maximum Sensitivity Substrate (Thermo Fisher Scientific, Waltham, MA, USA) in a ChemiDoc touch image system (Bio-Rad Laboratories, Tokyo, Japan). Quantification of the results was performed by densitometry using Image Lab Software version 5.2 (Bio-Rad Laboratories, Tokyo, Japan).

### 2.7. Statistical Analyses

Values are presented as mean ± S.D. Statistical significance of differences was assessed using Student’s *t*-test or one-way ANOVA followed by Dunnett’s post hoc test for multiple comparisons. Statistical significance was set at *p* < 0.05.

## 3. Results

### 3.1. IQOS Aerosol Exposure Increased TBA-RS and GSH Concentrations in Lung Tissue

**Figure 1:**
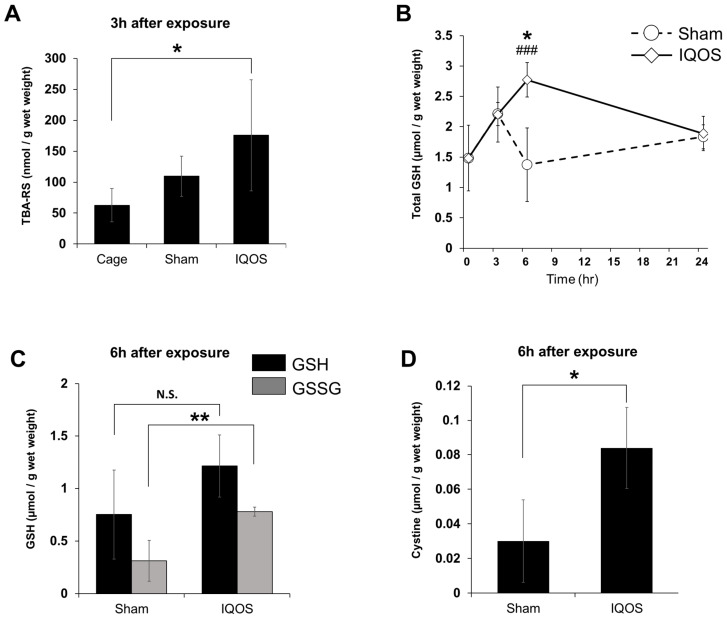
TBA-RS and GSH levels in the whole lungs of mice exposed to IQOS. (**A**) The TBA-RS concentration in lung tissue of mice 3 h after exposure to IQOS, sham controls, and cage controls. (**B**) Total GSH concentrations in the lung tissue of mice are shown at each hour after IQOS exposure. * *p* < 0.05 versus IQOS 6 h exposure group. ### *p* < 0.001 versus 0 h group. (**C**) The glutathione concentration of reduced form (GSH) and oxidized form (GSSG) in lung tissue of mice 6 h after exposure to IQOS or sham controls. (**D**) Cystine concentration in lung tissue of mice 6 h after exposure to IQOS or sham controls. * *p* < 0.05, ** *p* < 0.01. N.S.: not significant. N = 3.

**Figure 2:**
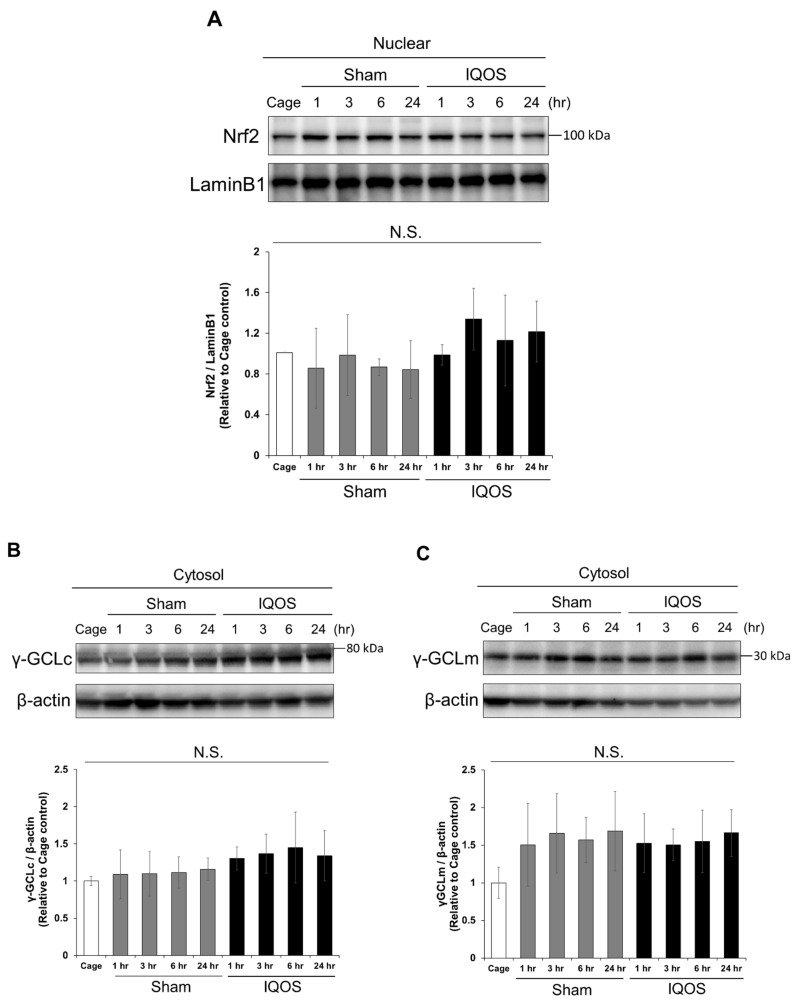
Nrf2 and GCL expression levels in the whole lungs of mice exposed to IQOS. (**A**) Analysis of nuclear Nrf2 expression in the lungs of murine cage controls, sham controls, and at various times after exposure to IQOS. The density of Nrf2 bands were measured, and the ratio of expression normalized to LaminB1 was calculated and expressed as fold-change relative to cage control mice. (**B**) Expression analysis of γ-GCLc in the cytoplasmic fraction of the lungs of murine cage controls, sham controls, and at various times after exposure to IQOS. The density of γ-GCLc bands were measured, and the ratio of expression normalized to β-actin was calculated and expressed as fold-change relative to cage control mice. (**C**) Expression analysis of γ-GCLm in the cytoplasmic fraction of murine lungs from cage controls, sham controls, and at various times after exposure to IQOS. The density of γ-GCLm bands were measured, and the ratio of expression normalized to β-actin was calculated and expressed as fold-change relative to cage control mice. N.S.: not significant. N = 3.

First, we examined whether IQOS aerosol exposure altered the TBA-RS concentrations in the whole-lung tissue. TBA-RS concentrations in the IQOS-exposed group were significantly higher than those in the sham control group 3 h after exposure to aerosols (Figure 1A). As IQOS aerosol exposure caused an oxidative stress response in whole-lung tissue, we examined GSH and cystine concentrations in the whole-lung tissue using HPLC-FL. The IQOS-exposed group showed a significant increase in total GSH concentration 6 h after exposure compared to the sham control group (Figure 1B). Although there were no significant differences in the concentration of reduced GSH in the whole lung tissue of the IQOS-exposed group compared to the sham control group 6 h after exposure, the concentration of GSSG was significantly increased in the IQOS-exposed group (Figure 1C). In addition, the cystine concentration in whole-lung tissue significantly increased 6 h after exposure to IQOS aerosols (Figure 1D). The intracellular GSH concentration is regulated by the transcriptional factor, Nrf2. Therefore, we examined the nuclear accumulation of Nrf2 and the expression levels of γGCLm and γGCLc, the rate-limiting enzymes for GSH synthesis, in the lungs of mice exposed to IQOS aerosols. As a result, Nrf2 accumulation and related protein expression levels were unchanged in the nuclear and cytosolic fractions of IQOS-exposed lungs (Figure 2A–C).

### 3.2. IQOS Aerosol Exposure Activated the eIF2α-ATF4 Pathway

**Figure 3:**
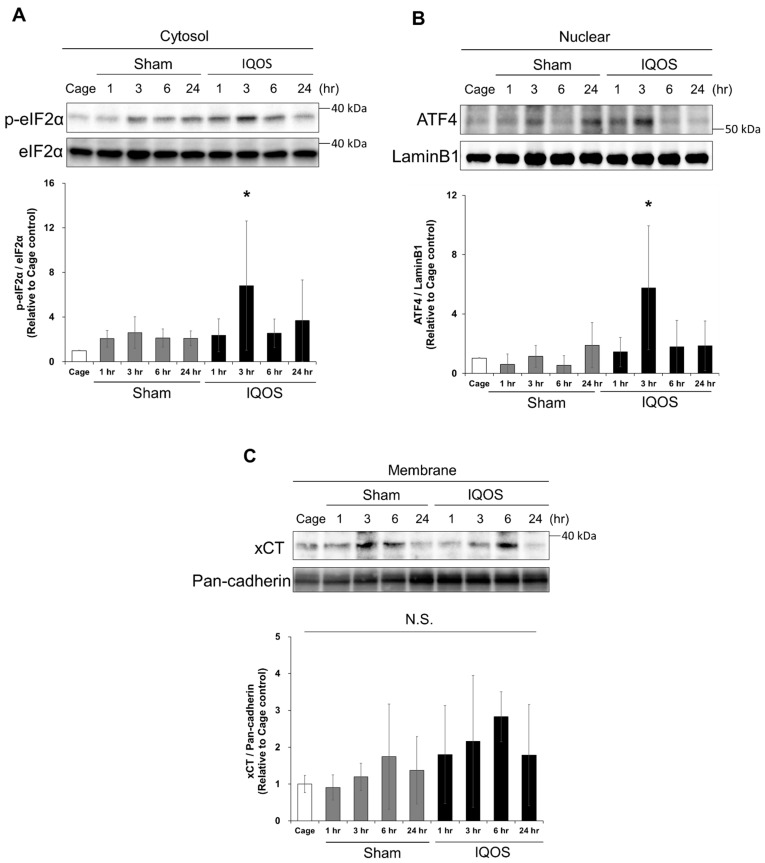
Effects of IQOS exposure on the eIF2α-ATF4 axis in murine whole-lung tissue. (**A**) Expression analysis of p-eIF2α and eIF2α in the cytoplasmic fractions of the lungs from murine cage controls, sham controls, and at various times after exposure to IQOS. The densities of p-eIF2α bands were measured, and the expression ratio to eIF2α was calculated and expressed as a normalized fold-change relative to cage control mice. (**B**) The analysis of nuclear ATF4 expression in murine lungs of cage control, sham control, and various times after exposure to IQOS is shown. The density of ATF4 bands were measured, and the expression ratio normalized to LaminB1 was calculated and expressed as fold-change relative to cage control mice. (**C**) Expression analysis of xCT on cell membranes in the murine lungs of cage controls, sham controls, and at various times after exposure to IQOS. The density of xCT bands were measured, and the expression ratio normalized to Pan-cadherin was calculated and expressed as fold-change relative to cage control mice. * *p* < 0.05 versus cage control group. N.S.: not significant. N = 3.

The present study suggests that exposing mice to IQOS aerosols induces oxidative stress in the lung tissue. Oxidative stress and ER stress are closely linked in mammalian cells [18]. Cigarette smoke exposure induces ER stress in small airway epithelial cells and alveolar epithelial cells [19,20]. However, whether exposure to IQOS aerosols induces ER stress in lungs remains unknown. Therefore, we examined the phosphorylated eIF2α (p-eIF2α)/eIF2α ratio and accumulation of ATF4 in the lungs of IQOS-exposed mice by western blot. The phosphorylation of eIF2α in the cytosolic fraction of the IQOS-exposed group was more significant than that of the sham control group 3 h after exposure to aerosols (Figure 3A). Furthermore, exposure to IQOS aerosols for 3 h significantly induced the accumulation of ATF4 in the nucleus (Figure 3B). Both increases in cytosolic p-eIF2α and the accumulation of nuclear ATF4 returned to the basal state 6 h after exposure to IQOS aerosols. Next, we examined the expression level of xCT, a cystine transporter, in the membrane fraction of lung tissue by Western blot (Figure 3C). The results showed an increasing tendency of xCT protein expression 3–6 h after exposure to IQOS aerosols. These results suggest that cystine influx into cells in response to oxidative stress from IQOS aerosol exposure resulted in a rapid increase in GSH production and protection by GSH. Furthermore, since cigarette smoke exposure is known to cause hypoxic injury, we examined nuclear accumulation of HIF1α protein by Western blot. However, IQOS aerosol exposure did not induce HIF1α accumulation in the nucleus (data not shown). These results indicate that ER stress is transiently induced in the lungs by short-term exposure to IQOS aerosols.

### 3.3. IQOS Aerosol Exposure Induced the Accumulation of Truncated HO-1 in the Nuclear Lung Tissue

**Figure 4:**
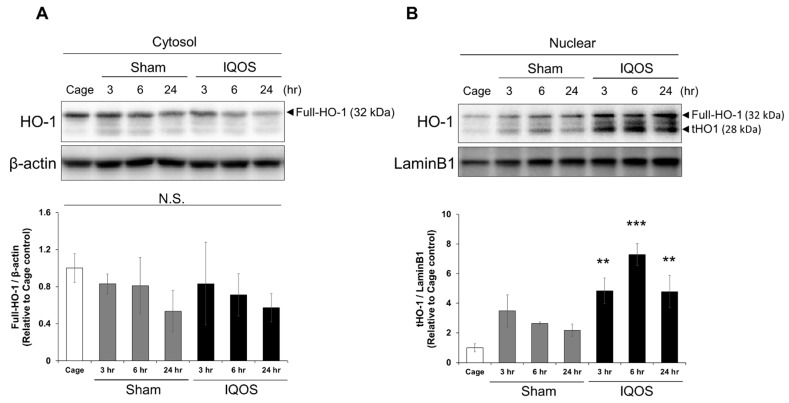
Alterations in HO-1 protein localization in murine lungs by exposure to IQOS. (**A**) Expression analysis of HO1 in the cytoplasmic fraction of murine lungs from cage controls, sham controls, and at various times after exposure to IQOS. The density of HO-1 bands was measured, and the expression ratio normalized to β-actin was calculated and expressed as fold-change relative to cage control mice. (**B**) Expression analysis of nuclear HO-1 in the lungs of at cage controls, sham controls, and at various times after exposure to IQOS. The density of HO-1 bands was measured, and the expression ratio normalized to LaminB1 was calculated and expressed as fold-change relative to cage control mice. ** *p* < 0.01, and *** *p* < 0.001 versus cage control group. N.S.: not significant. N = 3. Full-HO-1: full-length HO-1; tHO-1: truncated HO-1.

HO-1, a well-recognized antioxidant factor, is truncated and translocated into the nucleus in response to oxidative stress [21]. However, no reports have evaluated the response of HO-1 to IQOS aerosol exposure. Therefore, we detected the accumulation of truncated HO-1 in the lung nuclear fraction by Western blot to evaluate the stress response in mouse lung tissues more broadly due to IQOS aerosol exposure. The accumulation of full-length HO-1 (32 kDa) and truncated HO-1 (28 kDa) in the nucleus was significantly increased in the lung nuclear fractions of the IQOS-exposed group compared to the sham control group (Figure 4A). However, there were no significant differences in the expression levels of cytoplasmic HO-1 proteins between the IQOS-exposed and the sham control group (Figure 4B). These results indicate that the truncation of HO-1 protein and its translocation into the nucleus are dependent on some types of stress induced by IQOS aerosol exposure.

## 4. Discussion

Because it takes a long time for health effects to become apparent, epidemiological data may not be reported for many years. In contrast, in vivo studies can clarify the biological effects of the short- and/or long-term exposure of chemicals. However, there have been few reports on the biological effects of HTPs, owing to the lack of standardized methods for exposing animals to HTP aerosols. Hence, we recently developed an animal exposure apparatus to allow for animals to inhale mainstream aerosols from HTPs to investigate the biological effects of IQOS [14]. While long-term effects, such as genotoxicity and carcinogenesis, are important to study, it is also crucial to evaluate acute effects, such as stress responses.

In our previous in vitro study, we found that GSH was rapidly decreased in lung epithelial cells upon exposure to the aerosol extract of HTPs [13]. Moreover, a recent in vivo study, using our developed apparatus for aerosol exposure in animals, revealed that the reduced form of GSH decreased in mouse lung macrophages [15]. These results indicate that GSH was consumed and transiently decreased during the detoxification of xenobiotics, such as aldehydes from HTP aerosols.

In this study, we conducted an in-depth examination of the specific responses induced by IQOS aerosol exposure in an in vivo system, based on changes at the molecular level, such as transcription factors. Considering the effects of restraint stress, we used three groups of mice (mice continuously exposed to five IQOS heatsticks, sham control mice that were exposed to air using the same device, and cage control mice for which exposure manipulation was not performed) to evaluate acute responses in lung tissue within 24 h of IQOS aerosol exposure. Three hours after exposure, there was a significant increase in TBA-RS, a marker of oxidative stress, and a significant increase in total GSH concentration in the lung was subsequently observed. Thus, we focused on the acute stress responses induced by IQOS aerosols, and the related factors were comparatively analyzed in the present study. GSH levels transiently decrease during reaction with xenobiotics to form conjugates, or the reduced form of GSH is oxidized to GSSG by reaction with reactive oxygen species in the cell [13,15]; however, the biosynthetic system and redox system of GSH are rapidly activated in response to the decrease in the reduced form of GSH [22,23]. The Nrf2 pathway is a well-known transcription factor involved in GSH synthesis [24,25]. Hence, when these alterations were investigated, the nuclear translocation of Nrf2 due to IQOS aerosol exposure was not observed. Therefore, in the present study, we focused on ATF4 [26,27], a known transcription factor that induces the expression of genes involved in intracellular GSH synthesis and its metabolism. As such, a significant accumulation of ATF4 was found in the nuclear fraction of lung tissue 3 h after IQOS exposure. Next, the concentration of cystine, its transporter xCT, and the rate-limiting enzyme, GCL (GCLm, GCLc), were measured as factors of the GSH biosynthetic system in which ATF4 is involved. The results revealed a significant increase in cystine concentration in the lung tissue six hours after IQOS exposure, and an upward trend in xCT expression levels in the lung plasma membrane fraction after IQOS aerosol exposure. However, no significant difference in GCL expression was observed at the protein level. Therefore, we interpreted that a significant change in ATF4 3 h after IQOS exposure, followed by an increase in total GSH concentration 6 h after exposure, as result of the activation of GSH biosynthesis in this study. It is likely that ATF4-related GSH biosynthesis is partially dependent on the increased cystine uptake in lung tissues.

Interestingly, an examination of HO-1, a stress-response protein whose expression is partially regulated by ATF4 [28], revealed that, despite the absence of nuclear changes in Nrf2 and HIF-1 following IQOS aerosol exposure, truncated HO-1 translocation and accumulation were observed in the lung nuclear fraction after exposure. This change in intracellular expression has also been reported in exposure to conventional cigarette smoking [29,30], indicating that IQOS induces the same biological response as combustible cigarettes. Moreover, analysis of the upstream ATF4 regulatory system revealed that the phosphorylation of eIF2α was enhanced in the lung cytoplasmic fraction after exposure to IQOS aerosols. These results indicate that ER stress, accompanied by activation of the eIF2α-ATF4 axis, is induced as an early response to IQOS aerosol exposure.

In the present study, based on the analysis of transcription factors, no change was observed in the expression of HIF-1a. Thus, hypoxic stress was not induced by exposure to IQOS in mice with our developed device. Furthermore, the results suggest that IQOS exposure induced immediate GSH oxidation and the subsequent activation of GSH synthesis might be dependent on ATF4, but not Nrf2. In addition, our study is the first to demonstrate that IQOS exposure activates the eIF2α-ATF4 axis (an ER stress response) and induces the nuclear translocation of truncated HO-1 in lung tissue. In fact, given that studies have reported that combustible cigarette smoking induces ER stress, the changes found in murine lungs in this study demonstrate that inhalation of IQOS elicits a biological response similar to that of combustible cigarettes [19,20]. Moreover, by conducting a comparative study using a sham control, it was possible to eliminate the effects of restraint stress, etc.

A recent in vivo study demonstrated that irritating and carcinogenic compounds including aldehydes and polycyclic aromatic hydrocarbons are contained in the IQOS mainstream and indicates that IQOS causes grave lung damage and promotes factors that increase cancer risk [31]. Meanwhile, a recent review suggests that HTPs may be products with a reduced risk of chronic diseases, including respiratory and cardiovascular diseases and cancer, compared to traditional smoking, although exposure to IQOS has been reported to alter mitochondrial function, which may further exaggerate airway inflammation, airway remodeling and lung cancer [32]. However, the effect of composition in HTP on lung cancer development is unknown and experimental evidence is very limited, making it difficult to address the possibility of carcinogenesis from HTP use at this time. Therefore, further studies on long-term exposure to IQOS and other HTPs are needed in the near future to understand the true mechanisms of HTPs in lung cancer formation.

## 5. Conclusions

Oxidative stress induced by IQOS aerosol exposure resulted in enhancement of cystine influx and GSH biosynthesis in mouse lung tissue. Moreover, IQOS exposure activated the eIF2α-ATF4 axis and induced nuclear translocation of truncated HO-1 in lung tissue. These significant changes at multiple molecular levels observed after transient exposure of IQOS aerosols provide useful evidence that the biological effects of HTPs, such as IQOS cannot be ignored regarding the damage it causes to the lungs.

## Data Availability

Data is contained within the article.
